# The Role of Adipocytes Recruited as Part of Tumor Microenvironment in Promoting Colorectal Cancer Metastases

**DOI:** 10.3390/ijms25158352

**Published:** 2024-07-30

**Authors:** Yunxia Ma, Miljana Nenkov, Yuan Chen, Nikolaus Gaßler

**Affiliations:** Section Pathology of the Institute of Forensic Medicine, Jena University Hospital, Friedrich Schiller University Jena, Am Klinikum 1, 07747 Jena, Germanymiljana.nenkov@med.uni-jena.de (M.N.);

**Keywords:** obesity, adipose tissue, adipocyte–mesenchymal transition, CAAs, TME, microbiota, colorectal cancer metastases, therapeutics

## Abstract

Adipose tissue dysfunction, which is associated with an increased risk of colorectal cancer (CRC), is a significant factor in the pathophysiology of obesity. Obesity-related inflammation and extracellular matrix (ECM) remodeling promote colorectal cancer metastasis (CRCM) by shaping the tumor microenvironment (TME). When CRC occurs, the metabolic symbiosis of tumor cells recruits adjacent adipocytes into the TME to supply energy. Meanwhile, abundant immune cells, from adipose tissue and blood, are recruited into the TME, which is stimulated by pro-inflammatory factors and triggers a chronic local pro-inflammatory TME. Dysregulated ECM proteins and cell surface adhesion molecules enhance ECM remodeling and further increase contractibility between tumor and stromal cells, which promotes epithelial-mesenchymal transition (EMT). EMT increases tumor migration and invasion into surrounding tissues or vessels and accelerates CRCM. Colorectal symbiotic microbiota also plays an important role in the promotion of CRCM. In this review, we provide adipose tissue and its contributions to CRC, with a special emphasis on the role of adipocytes, macrophages, neutrophils, T cells, ECM, and symbiotic gut microbiota in the progression of CRC and their contributions to the CRC microenvironment. We highlight the interactions between adipocytes and tumor cells, and potential therapeutic approaches to target these interactions.

## 1. Introduction

Colorectal cancer (CRC) is the third most commonly diagnosed malignancy and the second leading cause of cancer-related death in 2020 worldwide [1]. From 1992 to 2019, approximately 82.6% of patients died due to metastatic cancer, and colorectal cancer metastases (CRCM)-related deaths accounted for 7.4% of all deaths in the USA [2]. In CRC, around 60% of the patients ultimately develop distant metastases [3]. CRC cells invade the mucosal layers and move to the blood or lymphatic vessels, which is the first step in initiating the subsequent invasion–metastasis cascade [4]. CRC cells can also metastasize to the peritoneum via the peritoneal fluid. Liver, lung and peritoneal cavity are the most common sites of CRCM [5].

Epidemiological data indicate that obesity is independently and positively associated with CRC risk [6,7,8]. Obesity-related visceral white adipose tissue (vWAT), which is in physical contact with colorectal serosa, is associated with CRCM and CRCM-related mortality [9]. Moreover, ectopic fat accumulation in the submucosal layer has been involved in the pathogenesis of inflammatory bowel disease (IBD), which increases the risk of developing colorectal neoplasia [10,11,12,13]. Accumulated genetic and epigenetic changes are the main causes for CRC initiation. When CRC cells grow beyond the mucosal inner layer and further penetrate the muscle layers, they migrate/invade the resident submucosal vWAT and serosa vWAT. During tumor reprogramming, adipose tissue components are recruited into the tumor microenvironment (TME) and secrete a series of inflammatory adipokines that are essential to provoke a pro-inflammatory, pre-metastatic TME to promote CRC progression and metastasis [14,15]. Adipocytes undergo serial metabolic changes, especially lipolysis, to fuel tumor cells, meanwhile, adipocytes experience mesenchymal transition into fibroblast-like precursor cells, termed cancer-associated adipocytes (CAAs) [16]. In addition, the TME is infiltrated with a large number of pro-inflammatory immune cells, especially macrophages, T cells, and neutrophils, which are recruited from adipose tissue and blood through upregulated inflammatory factors from the obese TME, such as TGF-β1, IL-6, TNF-α, MCP-1, and so on [17]. Enhanced inflammation facilitates tumor progression and metastasis [18]. Those soluble secretions from the TME are also strongly involved in ECM remodeling by dysregulated ECM proteins and cell surface adhesion molecules, which increase contractibility between tumor and stromal cells; enhanced ECM remodeling promotes epithelial-mesenchymal transition (EMT) which EMT increases tumor migration and invasion into surrounding tissues or vessels and accelerates CRCM [19,20,21]. Moreover, the colorectal symbiotic microbiota is an active player, its profiles and metabolites also play an important role in the promotion of CRCM [22].

In this review, we summarize the current research focusing on the existing connection between adipose tissue and CRC, with a particular emphasis on the role of adipocytes, macrophages, neutrophils, T cells, ECM, and symbiotic gut microbiota in CRC progression and their contributions to CRCM. Specifically, we highlight the interactions between adipocytes and tumor cells found within the tumor microenvironment (TME), and potential therapeutic approaches to target these interactions.

## 2. Adipose Tissue, Obesity and Colorectal Cancer

Obesity is considered as adipose tissue dysfunction due to excessive fat accumulation which is associated with type II diabetes mellitus and insulin resistance-related metabolic disorders, including cancer [23].

### 2.1. Adipose Tissue, Type

**Figure 1:**
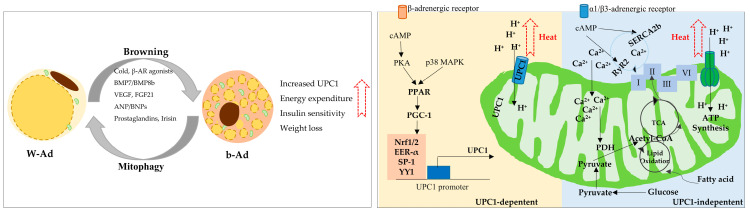
UPC1-dependent and -independent thermogenesis in WAT browning. WAT browning is stimulated by cold exposure, β-adrenergic receptor agonists, exercise, dietary chemicals (bile acid, sesamol, curcumin, and fish oil), and endocrine signals including irisin, bone morphogenetic protein 7 (BMP7), fibroblast growth factor 21 (FGF21) and prostaglandins [28,29]. WAT browning is characterized by increased mitochondrial numbers and upregulation of thermogenesis-related genes, especially mitochondrial uncoupling protein 1 (UCP1), which is the mechanistic component of heat production in classical BAT [30]. UCP1 expression is transcriptionally upregulated by peroxisome proliferation-activated receptor (PPAR)-coactivator-1α (PGC-1α), which is activated by cAMP-dependent protein kinase A (cAMP-PKA) and p38 mitogen-activated protein kinase (MAPK) [31]. In addition, activated α1/β3-adrenergic receptor signal stimulation can stimulate UCP1-independent thermogenesis via enhanced Ca^2+^ cycling, which is regulated by cAMP-dependent activation of sarco/endoplasmic reticulum Ca^2+^-ATPase2b (SERCA2b) and Ca^2+^ release channel ryanodine receptor 2 [32]. Increased Ca^2+^ concentration in mitochondria activates pyruvate dehydrogenase phosphatase (PDH) and ATP production [33]. An UCP1-independent mechanism in b-Ad can largely utilize glucose and improve glucose tolerance. Moreover, the trans-differentiation from beige to white adipocytes can be reversed via mitophagy [34,35].

Adipose tissue is classified into white adipose tissue (WAT) and brown adipose tissue (BAT). WAT, the predominant type of adipose tissue, mainly composed of white adipocytes (W-Ads), is accountable for lipid storage, whereas BAT cells, mainly containing brown adipocytes (B-Ads), are responsible for thermogenesis in mitochondrial uncoupling protein 1 (UPC1)-dependent manner [24]. Under normal conditions, these two cell types with opposing roles are essential for body homeostasis. In addition, W-Ads possess extraordinary plasticity and experience dramatic genetic, morphological, and functional changes in response to changes in temperature and nutritional state [25]. During cold exposure or the stimulation of β-adrenergic receptor (β-AR) agonists, a group of brown-like adipocytes, termed beige or brite adipocytes (b-Ads), are recruited within WAT depots to produce heat in a mitochondrial UPC1-dependent and -independent manner [26] (Figure 1). Both BAT activation and WAT browning lead to increased energy expenditure and protect against the development of obesity and type II diabetes mellitus [27].

### 2.2. Obesity, Systemic Dysregulated Adipokines, and Colorectal Cancer

WAT is the most dynamic component of body mass and varies from 3% to 70% of total body weight [36]. WAT depots are distributed in subcutaneous, breast, bone marrow, intramuscular, pericardial, and intra-abdominal regions (omentum, peritoneum, visceral) [37]. In a lean or healthy body, subcutaneous white adipose tissue (scWAT) is the prominent AT depot, accounting for approximately 80% of all adipose tissue. When adipose tissue expansion exceeds the storage capacity of scWAT, fat is delivered to the vWAT, which is linked to overweight (body mass index: BMI 25 to < 30) and obesity (BMI ≥ 30) [38]. The vWAT and submucosal WAT are the main types of adipose tissue that are in physical contact with the colon or rectum. Fat deposits in the middle submucosal layer, also known as the fat halo sign, demonstrated on abdominal CT scans, can be found in patients with chronic IBD, such as Crohn’s disease and ulcerative colitis, overweight or obese patients, and other chronic diseases. Compared to the small intestine, the fat halo sign has been more frequently observed in different segments of the large intestine, mostly in the colon, and contributes to the pathogenesis of IBD [10,11,12,39]. IBD patients with severe inflammation show a higher risk of colorectal neoplasia including colorectal dysplasia and CRC [13,40]. vWAT is associated with insulin resistance and increased insulin-like growth factor-1 (IGF-1) levels, which in turn activates insulin/IGF signaling and can enhance fat collection by impairing glucose/lipid metabolism in CRC [41,42,43]. Higher vWAT is an independent predictive biomarker for poor clinical outcomes in metastatic CRC patients who underwent Bevacizumab treatment [44]. Using deep learning, it has been found that poorly differentiated tumor cell clusters adjacent to adipose tissue predict unfavorable clinical outcomes in CRC [45]. Interestingly, obese patients diagnosed with advanced CRC show that obese patients have a longer duration of chemotherapy and better prognosis compared to the control group [46]. However, obese colon cancer patients with stage II-III have higher rates of failed treatment and more recurrences [47]. In all, vWAT, as a potential prognostic marker, evaluation of the status of WAT, including type and distribution, before cancer treatment might be helpful in deciding the treatment strategy and predicting the efficacy.

Adipose tissue, as an active endocrine organ, secreting diverse adipokines, chemokines, and cytokines, not only induces local inflammation but also provokes systemic inflammation throughout the body via blood circulation. Increased serum levels of leptin, visfatin, tumor necrosis factor-α (TNF-α), and interleukin-6 (IL-6) in overweight/obese school-aged children (5–18 years) are associated with systemic inflammation, with upregulated blood counts of leukocytes, lymphocytes, platelets, and C-reactive protein [48,49]. Neutrophils have been described as an important player in maintaining systemic inflammation in obesity [50]. Elevated blood neutrophils are significantly correlated with higher BMI, and weight loss reverses the blood neutrophil-related pro-inflammatory effects by upregulation of neutrophil-activating peptide (ENA-78), downregulation of ROS production and cytokine release (IL-1β, IL-6, and TNFα) [51,52]. High circulating retinol-binding protein 4 (RBP4) and neutrophil gelatinase-associated lipocalin (NGAL) are correlated with micro- and macroalbuminuric diabetic groups [53]. The plasma level of IL-6 is 50% higher in the portal vein than in the peripheral artery blood of obese subjects, suggesting that vWAT is an important source of IL-6 secretion [54]. The anti-cancer factor, adiponectin is negatively correlated with intra-abdominal fat mass, especially vWAT, but not scWAT [55,56]. Lower levels of adiponectin are associated with higher levels of neutrophil-secreting CXCL8 in obese patients, indicating that adiponectin is negatively correlated with blood neutrophils in obesity cases [57]. Moreover, fatty acid binding protein 4 (FABP4) secreted by adipose tissue enhances tumor stemness and aggressiveness by activating the IL-6/STAT3/ALDH1 axis in breast cancer, suggesting that plasma FABP4 is a new link between obesity and cancer risk [58]. Therefore, the data suggest that those dysregulated circulating blood mediators-induced systemic inflammation are potential targets for obesity to reduce obesity-related cancer risk.

### 2.3. WAT Browning and High Grade of Colorectal Cancer

As mentioned previously, higher vWAT is a prognostic marker for metastatic CRC. Higher expression of UCP1 and lipolysis key enzyme hormone-sensitive lipase (HSL) are observed in peritumoral WAT of high-grade CRC samples than in low-grade CRC samples, indicating that increased WAT browning and lipolysis are required for CRC progression in adipocytes-rich TME [59]. Migration and invasion inhibitory protein (MIIP) is reversely correlated with WAT browning; knockdown of MIIP intensifies adipocytes browning and increases lipolysis in CRC cells [59]. WAT browning is also a key feature of cancer-associated cachexia (CAC) [60]. In vitro, exosomal miR-146b-5p derived from CRC tumor cells promotes WAT browning and causes cachexia, while the downstream target gene of miR-146b-5p, homeodomain-containing gene C10 (HOXC10) depletes the effects of miR-146-5p-induced WAT browning and improves cachexia [61]. Tumor-derived parathyroid-hormone-related protein (PTHrP) is essential for Lewis lung carcinoma (LLC)-associated adipose tissue browning and cachexia, and neutralization of PTHrP inhibits LLC-induced adipose tissue browning [62]. In vivo, increased systemic chronic inflammation and upregulated IL-6 levels in blood are observed in cachectic mice models. Compared to the control, silencing IL-6 shows smaller tumor weight, reduced UPC1 expression in scWAT, and rescued CAC phenotype in colon cancer model; blocking IL-6 by anti-IL-6 antibody results in a reduction of body fat and UPC protein level in scWAT of melanoma mice model [63]. WAT browning in peritumoral WAT and the whole body’s WAT is a potential prognostic marker for CRC and cachexia, inhibition of WAT browning is a promising approach for cancer treatment.

BAT activation plays an opposite role in thermogenic metabolism during tumor progression owning to differences in origin and distribution. UCP1-dependent BAT activation by cold exposure significantly inhibits tumor growth by reducing blood glucose levels and increasing glycolytic metabolism in CRC tumor-bearing mouse models [64]. Moreover, positron emission tomography–computed tomography (PET-CT) scanning shows that mild cold exposure significantly increases BAT activation and decreases the glucose uptake in. the tumor area in the patients with Hodgkin’s lymphoma compared to the healthy group [64]. BAT is a potential therapeutic target in cancer management.

## 3. Metabolic Adaption of Adipocytes to Cancer-Associated Adipocytes (CAAs) during Adipocyte Mesenchymal Transition

Accumulation of genetic and epigenetic changes initiates colorectal tumorigenesis and endows CRC cells with a powerful metabolic adaptive ability in response to diverse stresses, such as hypoxia, energy deprivation, and acidosis via conduction of the TME [65]. Adipocytes are a rich source of lipids for cancer cells. In an adipocyte-rich TME, adipocytes undergo a series of metabolic changes during tumor-derived metabolic reprogramming.

### 3.1. The Morphology and Source of Cancer-Associated Adipocytes (CAAs)

CAAs are a consequence of the interplay between adipocytes and tumor cells. CAAs are observed at the invasive tumor front in multiple cancer types, including breast cancer, ovarian cancer, pancreatic cancer, and CRC [16,66,67,68]. Clinical morphology of CAA exhibits a special slender phenotype, sometimes with mild atypia. CAAs are smaller than adipocytes from scWAT and vWAT [69]. Fluorescent lipid-treated adipocytes co-cultured with cancer cells show that fluorescent lipids exported by adipocytes are taken up by cancer cells, and increased lipolysis in adipocytes and increased adipogenesis and β-oxidation in cancer cells, meanwhile, cancer cells induce adipocyte adaption into CAAs with an altered phenotype: a fibroblast-like phenotype, due to dispersed lipid droplets [16,21]. This co-culture model reflects that increased lipolysis in adipocytes is the underlying mechanism of peritumoral WAT browning observed in advanced CRC samples. CAAs lose adipocyte differentiation markers, such as HSL, resistin, perilipin (PLIN), FABP4, adiponectin, and peroxisome proliferator-activated receptor *γ* (PPAR*γ*) [21]. In the light of these morphological changes, CAAs are also termed adipocyte-derived fibroblasts [70]. CAAs are broadly defined and can be transformed from different adipocyte lineages. CAAs are dedifferentiated from mature adipocytes, however, they can also be differentiated from adipose stem cells. In vitro and in vivo models indicate that mouse ASCs infiltrate the TME accompanied by alpha smooth muscle actin (αSMA) expression which is one of the major features of cancer-associated fibroblasts (CAFs) [19]. The co-culture of human ASCs and breast cancer cells showed that hASCs differentiated into myofibroblasts with the expression of myofibroblast markers, such as αSMA and tenascin-c, which promote breast tumor cell invasion [71]. In view of the fibroblast markers expressed in CAAs, CAAs are considered a source of CAFs, which play an essential role in colorectal metastasis [72].

### 3.2. Lipid Metabolic Adaption

#### 3.2.1. Lipolysis

**Figure 2:**
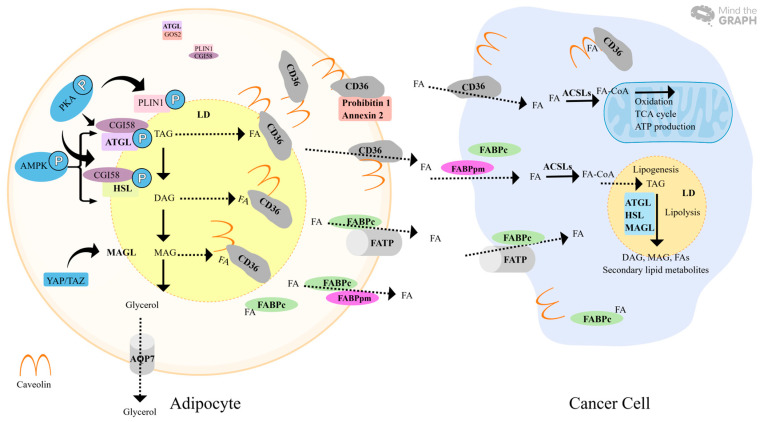
TAG lipolysis in adipocytes and FAs transportation in adipocyte and cancer cells. Activated ATGL, HSL, and MAGL are subsequently recruited into the LD to catalyze the conversion of TAG, DAG, and MAG into FAs and glycerol. Co-activators containing PLIN, CGI-58, and G0S2 are involved in channeling the lipolysis-related enzymes into the membrane of the LD. Activated PKA increases the activity of PLIN1, which leads to the dissociation of CGI-58 from PLIN1, and it further binds with activated ATGL and HSL for lipolysis. Phosphorylated AMPK upregulates ATGL and HSL. YAP/TAZ transcriptionally upregulates MAGL expression. Regarding the FA transportation, CD36-caveolin complex, and FABPc traffic the intracellular FAs to membrane, and further FFAs are exported by CD36, FABPpm, and FATPs. AQP7 facilitates glycerol flux across the membrane. TAG: triacylglyceride; DAG: diacylglycerol; MAG: monoacylglycerol; FAs: fatty acids; ATGL: adipose triglyceride lipase; HSL: hormonsensitive lipase; MAGL: monoacylglycerol lipase; PLIN1: perilipin 1; CGI-58: comparative gene identification-58 (CGI-58); G0S2: G0/G1 switch gene 2; PKA: protein kinase A; AMP-activated protein kinase: AMPK; YAP/TAZ: Yes-associated protein/transcriptional coactivator with a PDZ-binding domain; LD: lipid droplet; CD36: fatty acid translocase; FABP: fatty acid binding proteins; FABPc: cytosolic FABP; FABPpm: peripheral membrane FABP; FATP: fatty acid transport protein; AQP7: Aquaporin-7; ACSLs: long-chain acyl-CoA synthetases.

Triacylglycerides (TAGs) are the storage forms of fatty acids in adipocytes and are removed from adipocytes through lipolysis and mitochondrial oxidation. Three lipolysis-related enzymes composed of adipose triglyceride lipase (ATGL), HSL, and monoacylglycerol lipase (MAGL), and co-activators containing PLIN, comparative gene identification-58 (CGI-58), and G0/G1 switch gene 2 (G0S2), are involved in the lipolysis process (Figure 2).

In response to the stimulation of β-AR agonists, activated protein kinase A (PKA) induces the phosphorylation of PLIN1 and HSL, which leads to the dissociation of CGI-58 from PLIN1 and it further binds with activated ATGL and HSL for lipolysis [73]. Inhibition of PKA by PKA inhibitor (H89-2HCl) efficiently reverses the effects that colon cancer cells-derived conditioned medium induces lipolysis, thermogenesis, and cytokine secretion in adipocytes [59]. Activation of AMP-activated protein kinase (p-AMPK) upregulates ATGL and HSL expression [74]. PLINs are essential regulators of lipid droplets (LD) from a protective state to a lipolysis state. PLIN1 is predominantly present in adipocytes and is embedded in the phospholipid monolayer of LD [73]. PLIN-knockout mice show activated HSL, constitutively increased lipolysis, and a significantly reduced fat depot; depletion of PLIN reverses obesity by increasing the metabolic rate in obese mice [75]. PLIN2, a plasma biomarker, is potentially useful for the diagnosis of early-stage CRC [76]. CGI-58 interacting with ATGL facilitates lipolysis via induction of lipid hydrolase activity, while mutation of CGI-58, which is associated with Chanarin–Dorfman syndrome, leads to TAG accumulation through depletion of the interaction between PLIN and ATGL activity [77]. The mouse CGI-58 mutant S239E leads to the dissociation of the CGI-58/PLIN1 complex and activates ATGL, and increased solubility and stability are also observed in the CGI-58 mutant 3WA/S237E [78]. G0S2 is an endogenous inhibitor of ATGL. G0/G1 switch gene 2 (G0S2) diminishes the activity of ATGL via interacting with each other, and knockdown of G0S2 stimulates lipolysis via increased free ATGL [79].

Under the conduction of tumor cells, increased lipolysis is a prominent change during adipocyte mesenchymal transition. CRC displays increased lipogenesis, de novo lipogenesis, and upregulation of these process-related enzymes, whereas metastatic CRC shows upregulation of fatty acid oxidation-related enzymes [80,81]. Increased lipolysis-related enzymes are observed in aggressive cancers, suggesting that lipolysis is also acquired at high levels of malignancy. Compared to the normal colon tissue, cytosolic ATGL is significantly increased in colonic tumor tissue and augmented in obese colonic tumor tissue; ATGL promotes colon cancer cell migration and growth [82]. Elevated ATGL is significantly associated with CRC progression and is an unfavorable prognostic factor for CRC [83]. ATGL promotes proliferation via the upregulation of lipolysis in CRC [83]. Increased ATGL in aggressive breast cancer promotes invasiveness through the upregulation of pro-oncogenic lipid metabolites [84]. Additionally, lysine acetyltransferase 8 (KAT8) mediates de novo lipogenesis and lipolysis. KAT8 enhances lipolysis via upregulation of ATGL and HSL, which contributes to more invasive and migratory potential in CRC, and KAT8 acetylation reverses the promoting effect [85]. In pancreatic ductal adenocarcinoma (PDAC), disruption of the KRAS-HSL axis resulted in decreased lipid storage, tumor cell metabolic reprogramming, and reduced invasive and metastatic ability, indicating that the KRAS-HSL axis is a potential therapeutic target for PDAC [86]. Moreover, MAGL expression is elevated in many tumors. Elevated MAGL in colon cancer is associated with BMI, and silencing of MAGL inhibits cell proliferation and apoptosis via downregulation of cyclin D1 and B-cell lymphoma 2(Bcl-2) in CRC [87]. MAGL knockdown suppresses the expression of MMP14 in lung adenocarcinoma [88]. High MAGL levels are significantly associated with poor differentiation and a shorter survival time in hepatocellular carcinoma (HCC); MAGL knockdown inhibits apoptosis, proliferation, and invasion via the downregulation of lysophospholipids (PGE2 and LPA), indicating that secondary lipid metabolites (PGE2/LPA) regulated by MAGL are essential for tumor aggressiveness. In addition, the dysfunctional Hippo signaling pathway induces MAGL expression through the nuclear entry of YAP/TAZ in HCC [89].

Taken together, elevated ATGL, HSL, and MAGL and enhanced lipolysis are a common event in cancer, including CRC, especially aggressive CRC. Increased lipolysis produces rich FAs for oxidation and secondary lipid metabolites, which facilitate high levels of malignancy in CRC. Inhibition of lipolysis-related enzymes are a potential therapeutic target in advanced CRC.

#### 3.2.2. Lipid Transport

Free fatty acids (FFAs) influx/efflux across the membrane is mediated via CD36, caveolin, FABPs, and FATPs (Figure 2).

CD36, a major transporter protein expressed by adipocytes, is required for trafficking FFAs from adipose tissue [90]. Activated lipolysis increases caveolar endocytosis, CD36 deacylation, and CD36 dissociation from the interacting partners (prohibitin-1 and annexin 2), which contributes to the CD36-caveolar structure in the promotion of FFA mobilization from adipocytes into cancer cells [90]. Increased CD36 expression is enriched in metastatic sites of many cancer types, including CRCM, and CD36 silencing, or CD36-blocking antibodies significantly reduce metastasis [91]. In CRCM, increased CD36 expression induces lung colonization and orthotropic metastasis in mice through the CD36–MMP28–E-cadherin axis [91]. CD36-dependent metastasis is associated with unfavorable clinical outcomes in oral squamous carcinoma, lung squamous carcinoma, bladder cancer, and breast cancer [90]. Omental adipocytes co-cultured with ovarian cancer cells showed that apelin secreted from omental adipocytes activates the apelin/APL receptor (APJ) pathway, and further promotes ovarian cancer metastasis through the upregulation of CD36 by the APJ-STAT3 signaling pathway. Moreover, increased lipid uptake leads to enhanced lipid metabolism in ovarian cancer cells [92].

FABP has two distinct forms: FABPpm and FABPc. FABPc is involved in the transportation of intracellular FFAs to the membrane of adipocytes [93]. FABP4 is highly expressed in adipocytes, and depletion of FABP4 largely inhibits metastatic tumor growth in mice [15]. FABP5 promotes CRC tumor organoid growth via upregulation of hypoxia-inducible factor 1 (HIF-1); knockdown of FABP5 reverses the growth effects by downregulates the HIF-1α protein level and its downstream target genes, such as vascular endothelial growth factor (VEGF), carbonic anhydrase (CA9), pyruvate dehydrogenase kinase 1 (PDK1), BCL2 interacting protein 3 like (BNIP3L), and lysyl oxidase (LOX) [94]. FABP4 is lowly expressed in primary ovarian tumors, however, elevated FABP4 is observed in all omental metastases ovarian cancer samples; FABP4 is essential for ovarian cancer cells metastasizing to omentum via upregulation of lipid mobilization from omental adipocytes [16].

FATP takes up long-chain fatty acids (LCFAs) and activates them via acylation. However, the deletion of FATP1 not only leads to decreased FFA uptake but also impaired FFA efflux, suggesting that FATP1 could have a bidirectional function of FFA influx/efflux [95]. FATP2 is overexpressed in CRC and regulates fatty acid metabolism via communication with the PPARs pathway [96]. Overexpression of FATP1/4 increased fatty acid activation and uptake in adipocytes [97]. Knockdown of FATP1 and FATP4 independently leads to upregulation of PPARγ and CCAAT/enhancer binding protein α (C/EBPα) and reduced deposition of TAGs, DAGs, and MAGs [98].

Taken together, FA transport proteins are essential for importing the environment FFAs into cancer cells for lipid metabolites biosynthesis and energy production. Upregulated FA transport proteins are frequently observed in metastatic cancer. Targeting FA transport proteins has therapeutic potential for cancer by inhibiting FFA utilization.

#### 3.2.3. Enhanced Lipid Metabolism Mediated by Multiply Factors

During adipocyte mesenchymal transition, increased lipid metabolism is induced by hypoxia, hormones, growth factors, and inflammatory factors released from tumor cells as well as the tumor niche.

Hypoxia is associated with adipose tissue lipolysis and is the main driving force of cancer progression in solid tumors [99]. HIF-1 is the major regulator of oxygen homeostasis and HIF-1α is stabilized via interacting with co-activators in present to hypoxia [100]. High expression of HIF-1α is correlated with advanced-stage CRC and radio resistance in hyperglycemic rectal cancer, which predicts a poor clinical outcome in CRC patients [101,102]. Hypoxia plays a role in the regulation of lipid metabolism by upregulating lipogenesis-related genes (LPIN1, FASN, and SCD1) and FA transport protein (FABP1), and downregulating FAO-related genes, medium- and long-chain acyl CoA dehydrogenases (MCAD and LCAD); hypoxia promote cancer progression by inhibition of HIF-1-FAO/LCAD-PTEN axis; decreased LCAD is significantly correlated with late-stage HCC [103]. FABP5 has been identified as a critical HIF-1α binding partner; both FABP5 and HIF-1α are highly expressed and associated with poor survival in HCC [104]. Activated FABP5/HIF-1 pathways promote proliferation by upregulating lipid metabolism-related genes, such as lipid metabolism initiation gene (ACSL1), lipogenesis-related gene (GPAT, LPIN1, and DGAT2), FAO-related gene (carnitine palmitoyltransferase 1A, CPT1A), and lipolysis-related gene (ATGL) in HCC cells [104]. The FABP5-HIF-1 axis also promotes tumor spheroids’ growth of CRC cells [94]. Though targeting hypoxic HIF-1α is a promising approach for cancer treatment, the benefit may be in a context-dependent or tissue-specific manner. DNMT inhibitor (Zebularine) degrades HIF-1α and overcomes hypoxia-induced Oxaliplatin resistance by upregulating the activity of pyruvate dehydrogenase (PHD) that activated PHD increases the conversion from pyruvate to acetyl CoA for oxidation in CRC mice model [105].

Omental adipocytes secreting multiple adipokines, such as IL-8, IL-6, MCP-1, TIMP-1, and adiponectin, promote ovarian cancer cells to metastasize to the omentum via upregulation of lipolysis-related enzymes (PLIN and HSL) in omental adipocytes, FA transport protein (FABP4), and FAO-related enzymes (CPT1 and acyl-CoA oxidase 1) in cancer cells [16]. Blockage of IL-6 and IL-8 receptors, and their ligands by neutralizing antibodies reverses these promoted effects [16]. Leptin can both directly and indirectly upregulate lipolysis and FAO-related enzymes in adipose tissue through the sympathetic nervous system (SNS)/β-AR [106]. Surgical denervation of WAT inhibits leptin-stimulated lipolysis [107]. TNF-α, a lipid metabolism regulator, promotes lipolysis by downregulating PLIN in adipose tissue via several mechanisms, including the MAPK/ERK-PLIN, c-JNK-PLIN, and nuclear factor-κB (NF-κB)-PLIN/HSL pathways [108,109]. In the case of NF-κB inhibition, TNF-α-induced lipolysis is inhibited, suggesting that NF-κB is required to stimulate full lipolysis in response to TNF-α [108]. Moreover, TNF-α suppresses lipid uptake by downregulating FATP1, FATP4, and insulin signaling in adipocytes [95]. IL-6, mainly produced by stromal immune cells, induces lipolysis and β-oxidation in adipocytes from scWAT/vWAT [110]. IL-6 decreases the activity of lipoprotein lipase (LPL) in omental WAT and scWAT, indicating that IL-6 can reduce lipid uptake and deposition in adipose tissues [110]. In contrast, adiponectin plays a role in lipoprotein metabolism by increasing LPS to reduce TAG levels in patients with type II diabetes mellitus [111].

The expression and activity of lipolytic enzymes are regulated at the transcriptional and post-transcriptional levels by many transcription factors. It has been depicted that PPAR-γ eliminates the transcriptional repression by specificity protein 1 (Sp1) at the ATGL promoter and upregulates its expression in mature adipocytes [112]. Adipocyte-specific depletion of signal transducer and activator of transcription 5 (Stat5) leads to increased adiposity and decreased lipolysis via downregulation of ATGL and CGI-58 [113]. Fasting-induced interferon regulatory factor 4 (IRF4) increases lipolysis via upregulation of ATGL expression in insulin- and forkhead box O1 (FoxO1)-dependent pathways [114]. However, fat-specific protein 27 (FSP27) inhibits ATGL promoter activity by binding to early growth response protein 1 (Egr 1) [115]. Insulin suppresses lipolysis through the mTORC1-Egr1-ATGL and MAPK/ERK-ATGL axes in adipocytes [116,117]. Reduced ATGL expression is also observed in TNF-α-treated adipocytes [118]. Moreover, β-AR stimulation leads to increased activity of ATGL Ser (406), which is phosphorylated by PKA [119]. AMPK-ASKO mice have defective ATGL and HSL functions [79].

Therefore, a comprehensive understanding of the underlying mechanisms involved in lipolysis including inducer factors, signaling pathways, transcription factors, and downstream genes is needed to develop effective drugs targeting lipolysis for cancer therapy.

### 3.3. Glucose Metabolism Adaption, Lactate Production

Cancer cells prefer to utilize glucose for energy production and glycolytic intermediates to maintain cancer cells’ survival and proliferation, which is called aerobic glycolysis, also known as “The Warburg effect” [120]. Glucose uptake is limited by glucose transporters (GLUTs), which are regulated by HIF-1*α* and p38 MAPK pathways [121,122]. Enhanced glucose uptake in tumors contributes to T cell dysfunction via impaired glycolysis and interferon-γ (IFN-γ) production in T cells [123]. The competition for glucose uptake between cancer cells and immune cells, suggests that this competition could also occur in other stromal cells of the TME, such as adipocytes [123]. Hypoxia can upregulate GLUT-1 expression upon hypoxia treatment for 1 and 2 days in human normal adipocytes [124]. IFN-γ inhibits glucose uptake in adipose tissue and downregulates the GLUT-4 in cancer cachexia [125]. Overall, the data suggest that non-tumor-infiltrated adipocytes continue to take up glucose from the environment and lose the advantage of glucose uptake when infiltrated by tumor cells.

**Figure 3:**
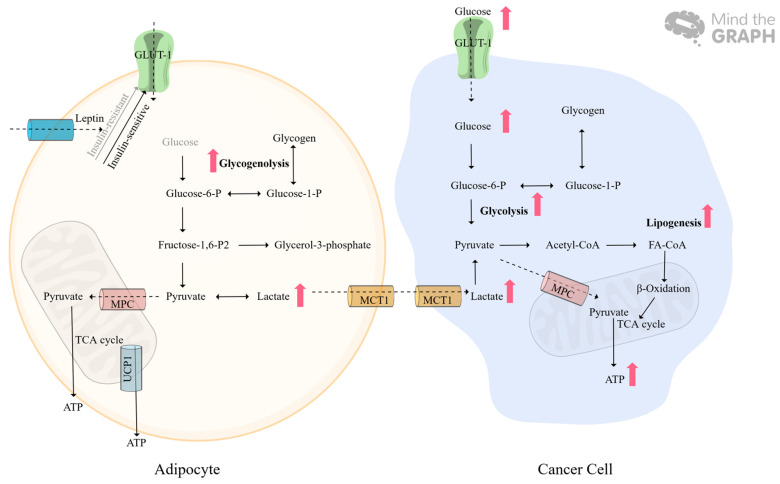
Glycogenolysis in adipocytes and glucose/lactate transport into tumor cells. Cancer cells are prior to utilize glucose from environment for glycolytic metabolism and lactate production, the so-called “The Warburg effect”. In obese, increased leptin inhibits GLUT-1 expression. Adipocytes use glycogen to produce more lactate. Lactate releases from adipocytes and is taken up into cancer cells via MCT1. Lactate converts into pyruvate, which further converts into acetyl-CoA for lipogenesis and oxidation. GLUT1: glucose transporter 1; MCT: monocarboxylate transporter; UPC1: uncoupling protein 1; MPC: mitochondrial pyruvate carrier; TCA cycle: tricarboxylic acid cycle; ↑: Increased.

Glycogen, as a carbohydrate storage molecule, is mainly found in the liver and skeletal muscle but is also found in adipose tissue. Glycogen changes dynamically during feeding and fasting, especially glycogen spikes during refeeding, suggesting that glycogen could be an energy sensor for the balance of glucose and lipid metabolism [126]. Accumulated polysaccharides as glycogen were the first observed in adipose tissue in the fasted-to-fed transition [127]. Since glycogen cannot pass through the plasma membrane, it can be used only through glycogenolysis. Glucose-1-phosphate (G1P), the direct product of glycogenolysis, can be freely interconverted with glucose-6-phosphate (G6P), which then enters glycolysis (Figure 3). In transgenic mice overexpressing the protein phosphatase-1 (PP1) glycogen-targeting subunit (PTG), stably elevated glycogen can be stored in adipose tissue compared to normal mice under fasting conditions; meanwhile, increased glycogen stimulates the secretion of leptin and lactate in adipose tissue [128]. Elevated leptin levels are associated with insulin resistance, which inhibits glucose uptake via impaired translocation of GLUT4 in the adipose tissue [129]. In addition, lactate influx and efflux are mediated by the monocarboxylate transporter (MCT). Adipose expansion-related hypoxia upregulates MCT1 and MCT4 in an HIF-1-dependent manner [124]. Lactate, as a danger signal, induces the expression of pro-inflammatory cytokines (CD11c, IL-1β, and TNF-α) and HIF-1α, which triggers the polarization of M1 macrophages [130]. Moreover, increased lactate creates an acidic TME that promotes tumor growth and metastasis in solid tumors, including CRC [131,132]. Lactate can be converted to pyruvate by lactate dehydrogenase (LDH). Mitochondrial pyruvate uptake via mitochondrial pyruvate carrier (MPC) is required to complete glucose oxidation, which is essential for UCP1-independent thermogenesis of BAT and WAT browning [133]. Ketone β-hydroxybutyrate (βHB) enhances mitochondrial respiration in an appropriate 91% of adipocytes and also accompanies increased adipose ATP production through upregulation of mitochondrial biogenesis and thermogenesis-related genes, such as PR/SET Domain 16 (PRDM16), Peroxisome proliferator-activated receptor gamma coactivator 1-alpha (PGC1α) and UPC1 [134]. Ketone βHB co-cultured with mouse intestinal tumor organoids strongly reduces the organoid’s growth, and ketogenic diets alleviate the tumor burden in a mouse model [135]. Moreover, the ratio of non-esterified fatty acids (NEFAs) to glycerol in transgenic mice indicates that increased TAG synthesis is promoted by glycogenolysis via potentially increased levels of glycerol-3-phosphate (G3P), which is converted from fructose 1, 6-bisphosphate (F1, 6BP) [136,137].

Taken together, tumor metabolic reprogramming increases glucose uptake from the TME, resulting in reduced glucose uptake in peritumoral adipocytes. Moreover, adipocytes convert glycogen into lactate via glycogenolysis. Lactate-production-related enzymes and inducers of UPC1-dependent thermogenesis offer therapeutic targets for the treatment of cancer metastasis.

## 4. Increased Inflammation via Recruitment of Immune Cells during Adipocyte–Mesenchymal Transition

During adipocyte–mesenchymal transition, secreted factors recruit and activate immune cells, such as macrophages, neutrophils, and T cells in the tumor niche, triggering invasion–metastasis processes through regulating the immune microenvironment [138]. All three types of immune cells have two subgroups: pro-tumor and anti-tumor, which are recruited through chemokine/chemokine ligands, colony-stimulating factor-1 (CSF-l)/CSF-1R, VEGF/VEGFR, and cytokines in tumor niche, such as TGF-β1, IL-6, TNF-α, MCP-1 (also known as CCL2), which are also upregulated in obese patient [139,140,141,142,143].

Macrophages are not only the resident cell type of adipose tissue but also important mediators of the innate immune response and adaptive immunity [144]. Macrophages circle damaged/necrotic adipocytes and form the crown-like structure (CLS), which induces a pro-inflammatory environment [145]. Omental macrophage CD68^+^ or CD163^+^ CLS status is associated with poor overall survival in advanced stages of ovarian cancer [145]. Anti-tumor macrophages (M1) can polarize into pro-tumor macrophages (M2) via IL-4, IL-10, and IL-13 [146]. The ratio of cancer-associated macrophages (CAMs) to M1/M2 varies up to the cancer type. High levels of CAMs-M1 are correlated with high Ki67 expression, and a high ratio of CAMs-M1/M2 is associated with an advanced grade of breast cancer [147]. However, a high content of CAMs-M2 is correlated with poor survival in CRC patients [148]. The same oxidative feature in CAMs-M2 and metastatic CRC suggests that CAMs-M2 are associated with metastatic CRC [149]. Unique CAMs-M2 interact with CRC cells via integrin-associated protein (IAP)/SIRPα signaling, promoting cancer cell growth and migration through the release of IL-4, IL-8, IL-10, and cysteinyl leukotriene D4 (LTD4), and increased IL-10 levels further enhance the function of CAMs-M2 [150,151]. The co-culture with CRC cells and CAMs-M2 leads to EMT and angiogenesis by activating the protein phosphatase of regenerating liver-3 (PRL-3)/MAPK/IL6 and IL-8 and NF-κB/VEGF-A pathways [152]. Moreover, CAMs-M2-derived IL6 promotes metastasis via upregulation of the JAK2/STAT3/miR-506-3P/FoxQ1 axis, which further leads to CCL2 release and CCL2-induced macrophage recruitment [153]. CAMs-M2-derived TGF-β1 triggers EMT progression through the TGF-β/Smad2/Snail pathway, and inhibition of the TGF-β/Smad2 pathway by the TGF-β receptor inhibitor suppresses EMT-promoting CRC metastasis [154]. CAMs-M2 are also significantly associated with increased VEGFR2 and HIF-1 expression; CAMs-M2 expressing VEGFR2 induces TGF-β1 through VEGF/VEGFR2 signaling in CRC [141]. In addition, high levels of TNF-α are significantly correlated with tumor grade, lymphovascular invasion, microsatellite instability status, and lymph node metastasis in CRC patients, and high TNF-α levels in stage II/III CRC patients indicate chemotherapeutic resistance to 5-Fluorouracil in CRC cells [155]. Therefore, CAMs-M2, as an essential component of TME, plays an important role in the regulation of cell proliferation, migration, invasion, metastasis, angiogenesis, ECM, EMT, and immunity in CRC.

At birth, the Treg cell ratio is almost the same in vWAT and scWAT; however, with long-term high-calorie feeding, Treg cells are reduced, and conventional T (Tconv) cells accumulate in vWAT [156]. In CRC, tumor-infiltrating lymphocytes are composed of anti-tumorigenic CD4+ (Th1 or Th22) and pro-tumorigenic CD4+ (Th17 or Tregs) cells, and the ratio of anti- and pro-tumor T cells is crucial for tumor progression [157]. CRC consensus molecular subtype 4 (CMS4) expresses high levels of Tregs, Th17, and myeloid-derived suppressive cells (MDSCs) and is associated with poor prognosis and drug resistance [158]. High levels of CD8+ cytotoxic T cells in the TME are correlated with improved prognosis in CRC [159]. However, the exclusion of CD8+ T cells is associated with a poor prognosis [160]. Effector T cells comprise a small population in CRC tumor samples. Increased recruitment of anti-inflammatory cells, such as CAFs, Tregs, CAMs, and MDSC, suppresses T cell function via the secretion of IL-10 and TGF-β [161].

Pro-tumor neutrophils (N2) infiltration is known to be the central contributor during the transformation from IBD to colitis-associated cancer by releasing IL-1β and IL-23 [162,163]. Neutrophil extracellular traps (NETs) and neutrophils are co-localized in both CRC and paired metastatic CRC [164]. Infiltrated neutrophils can also inhibit T cell function via MMP9 mediated TGF-β. Moreover, immune inhibitory receptors, such as PD-1, CTLA-4, TIM-3, and LAG-3, deactivate T cell function in CRC [165]. TGF-β, a rich cytokine in obese conditions, induces neutrophil infiltration by upregulation of TGF-β/Axl/CXCL5 signaling, resulting in the promotion of HCC progression [166]. Fibroblast growth factor 19 (FGF19) is strongly associated with CRC liver metastasis (CRLM) via activation of FGFR4/JAK2/STAT3 signaling-mediated hepatic stellate cells polarization to inflammatory CAFs, which further promote neutrophil infiltration and NETs formation for liver colonization of CRC cells; inhibition of neutrophil by neutralizing antibody blocks FGF19-mediated CRLM [167]. Elevated CD66b^+^CD16^+^ neutrophils (N2) and reduction of NK cells are observed in CRC tumor samples; the co-culture of CRC cells and immune cells indicates that neutrophils inhibit the anti-tumor activity of the NK cells and promote tumor growth by disturbing lipid raft formation-mediated by CD16/TAK1/NF-γB pathway; CD16 knockdown restores the immunosuppression in CRC patient-derived organoids [162]. Blood neutrophil to lymphocyte ratio (NLR) has been recognized as a biomarker for predicting the prognosis of solid tumors, and high NLR and IL-6 are significantly correlated with worse overall survival in non-metastatic CRC [163,168].

Therapeutic targeting of immune cells, including recruitment, soluble factors, factor-related receptors, and function-related pathways, offers a promising strategy for cancer treatment. For example, blocking CSF1R by PLX3397 effectively suppresses tumor growth and metastasis and sensitizes CRLM to 5-FU or anti-PD-1/-CTLA-4 [169]. Blocking TNF-α by a monoclonal antibody effectively inhibits tumor growth, increases tumor immunity, and decreases the member of CAMs-M2 in murine CRC cells-transplanted mouse model [170]. The TGF-β and JAK pathways play an important role in immune cell infiltration and are promising therapeutic targets for cancer treatment. YM101, a dual antibody targeting TGF-β and PD-L1 exhibits superior antitumor activity compared to monotherapy in mice bearing breast cancer [171]. Targeting FGF19-mediated FGFR4/JAK2/STAT3 signaling with an FGFR4 inhibitor (Fisogatinib) inhibits FGF19-induced CRCM and prolongs animal survival [172]. However, two resistant mutations in FGFR4 were detected after Fisogatinib treatment in HCC patients; LY2874455 designed targeting FGFR4 V550L can overcome Fisogatinib resistance in a mouse model [173]. A JAK inhibitor (Tofacitinib) leads to a broad inhibition of inflammation by interacting with multiple cytokine receptors for the treatment of IBD [174]. Tofacitinib improves the pathologic features of celiac disease by inhibition of IL-15 signaling in T3^b^-hIL-15 Tg mice; however, Tofacitinib shows a side effect with an accumulation of omental WAT and elevated lipids in ulcerative colitis patients, suggesting that the lipid profile should be monitored to avoid hyperlipidemia in patients receiving Tofacitininb therapy in the long-term [175,176].

## 5. Enhanced Extracellular Matrix Remodeling during Adipocyte–Mesenchymal Transition

ECM is an important non-cellular component in adipose tissue as well as in the TME. ECM is composed of numerous collagens, laminins, fibronectin, elastin, nidogen, and proteoglycans, and is a physical scaffold for supporting cellular activities, including proliferation, differentiation, and migration [177]. Excess ECM deposition resulting in increased fibrosis is observed in obese adipose tissue, as well as in multiple cancers, including CRC [14,178,179]. In addition, cell surface adhesion molecules, such as cadherins, integrins, and selectins, mediate cell-ECM interactions between contract cells and result in a mesenchymal transition that facilitates cancer invasion and determination of metastatic organotropism [180].

It has been reported that decellularized ECM isolated from obese mammary glands significantly increases breast cancer cell invasion compared with ECM isolated from lean mammary glands [178]. Upregulated collagen VI is observed in both obese and tumor ECM and is the major driver of cell adhesion and migration via NG2/EGFR and MAPK pathways. Moreover, inhibition of NG2/EGFR crosstalk and MAPK pathway reduces ECM-induced invasion [178]. Adipocyte–mesenchymal transition results in the formation of CAAs, which are accompanied by decreased E-cadherin and increased vimentin, collagen 1, MMP11, and PAI-1 levels [181]. CAAs and their secretions, such as adipokines, pro-inflammatory cytokines, and ECM molecules, are strongly involved in ECM remodeling, which is essential for promoting the cancer phenotype to a more aggressive mode. PAI-1 is involved in the regulation of adipose tissue biology, including insulin signaling, adipocyte differentiation, and inflammatory cell recruitment [182]. PAI-1 is secreted by both adipocytes and cancer cells; however, cancer cells express five times more PAI-1 than adipocytes [183]. In addition, cancer cells migrate faster in CAAs-embedded collagen gel than in mature adipocytes embedded in collagen gel, and CAAs-derived collagen remodeling is regulated by the PAI-1/PLOD2 axis [181]. PLOD2, a procollagen-lysine, 2-oxoglutarate 5-dioxygenase 2, is the key enzyme involved in ECM remodeling to form a stabilized collagen net. PLOD2 induces EMT and enhances cancer cell stemness and colonization by upregulating cytoplasmic succinate levels [184]. A higher level of type I collagen is significantly associated with CRCM compared to CRC without metastasis; and type I collagen treatment promotes CRC lung metastasis, as well as stemness via the collagen/integrin α2β1-PI3K/AKT and EMT pathways [179]. MMP3 enrichment in adipocyte-derived exosomes can improve the tumor-invasive ability of lung cancer metastasis by upregulating the activity of MMP9 [185]. The vasoactive peptide, apelin, an adipose factor, belongs to the adipokine family. Apelin-13 promotes proliferation via activation of the Jagged/Notch signaling pathway in colon carcinoma [186]. Apelin treatment not only increases the migrated capability through stimulation of protrusions formation and increased ratio of filamentous (F-actin) to monomeric (G-actin) and cofilin for cytoskeleton reorganization, but also enhances the invasiveness via upregulation of MT1-MMP (membrane-anchored MMP) for degradation and remodeling of ECM [187].

Increased fibroblastic markers such as SMA and fibroblast specific protein 1 (FSP-1) in CAAs, are considered one of the sources of CAFs. In addition to adipocytes, CAFs can be recruited from the mesenchymal lineage and multiple non-fibroblast lineage cells, including circulating fibrocytes, epithelial cells, endothelial cells, pericytes, and smooth muscle cells, which are also components of adipose tissue [188]. The transformation of CAFs is activated by tumor cell-derived soluble factors such as TGF-β, platelet-derived growth factor (PDGF), reactive oxygen species (ROS), and stromal-derived factor (SDF) [189]. Inflammatory cytokines, such as IL-1β, IL-6, and TNF-α, can also induce myofibroblast trans-differentiation and produce more matrix proteins and proteases [190]. It has been shown that a CAF-conditioned medium promotes CRC cell migration through the regulation of EMT-related markers, accompanied by reprogrammed lipid metabolism [191]. Active stroma with high levels of secreted TGF-β, pro-inflammatory factors, and a mesenchymal phenotype are characteristics of CMS4 in CRC. CMS4 has the worst overall survival compared to the other CMS subgroups [192]. Imatinib treatment shifts CMS4 to more CMS2 by increasing proliferation-related genes, including MKI67, WNT- and MYC-target genes, and KEGG cell cycle genes in primary colon cancer [193].

Moreover, ASCs, as progenitor cells of mature adipocytes, play a role in promoting tumor growth. High levels of leptin in obese ASCs are essential for inducing EMT and metastasis to the lung and liver via upregulation of the metastasis genes SERPINE1, MMP-2, and IL-6 [194]. Moreover, ASCs isolated from the abdominal adipose tissue of obese patients promote tumor invasion through increased ECM degradation-related proteins, such as calpain-4, calpastatin, and MMP15; depletion of those protease/protease inhibitors reverses the effects [195]. Integrin αVβ5 is the functional receptor of cysteine-rich 61 (Cyr61), which is an angiogenic inducer that is highly expressed in ASCs isolated from CRC patients compared to the healthy group. Cyr61 promotes vasculogenic mimicry formation and further accelerates CRC metastasis by activating the αVβ5/FAK/HIF-1α/STAT3/MMP2 and αVβ5/FAK/NF-ĸB pathways [196]. Moreover, the level of Cyr61 was positively correlated with the advanced TNM stages of CRC. Combination treatment with an integrin αVβ5 inhibitor and VEGF inhibitor (Bevacizumab) has a synergistic effect on the inhibition of CRC progression [196]. Blocking integrin α2β1 using an inhibitor (E7820) significantly enhances drug efficacy and inhibits CRCM [179].

Taken together, upregulated ECM proteins and adhesion molecules in tumor-adjacent adipose tissue promote ECM deposition and shape the TME into a more invasive mesenchymal phenotype via enhanced ECM remodeling and cell–ECM interactions.

## 6. The Role of Microbiota in Adipocyte-Rich Colorectal Cancer Metastases

The gut microbiota encompasses 100 trillion microorganisms that inhabit the gastrointestinal tract and play a crucial role in maintaining homeostasis and health [197,198]. Microbiota metabolites, including short-chain fatty acids (SCFAs), secondary bile acids, phenols, and ammonia, are major mediators of communication between the microbiota and host [199,200].

Dietary habits influence the composition of the gut microbiota and its ability to store dietary energy [199,201,202]. Furthermore, as observed in animal model studies, a maternal high-fat diet has been shown to significantly affect the gut microbiome of offspring, leading to a higher occurrence of metabolic syndrome accompanied by insulin resistance, elevated visceral fat, and adipocyte hypertrophy during the offspring’s lifetime [203,204]. Microbes, including Oscillbacter, Clostridiaceae, and Erysipelotrichaceae, were significantly differentially abundant in obese individuals compared to non-obese [205,206,207]. Additionally, the studies reported an increased ratio of Firmicutes/Bacteroidetes in obesity [208,209,210] while others claimed that this ratio was not always elevated in obesity [50,211]. Impairment of the gut microbiota composition in obesity causes the microbiome to switch to more efficient food digestion, resulting in higher food uptake and intestinal permeability [212]. This enables bacteria and bacterial products, such as LPS, to smoothly cross the intestinal barrier, triggering TLR4 myeloid differentiation primary response protein (MYD88), NF-κB signaling, and innate immune cell recruitment, leading to inflammation progression initiated by obesity [198,213,214,215]. This inflammatory process further reshapes the intestinal microbiome, contributing to elevated levels of harmful bacterial metabolites, including trimethylamine-N-oxide, secondary biliary acids, and amines, and lowering the content of protective SCFAs [198,216,217].

Bacterial species, including Fusobacterium nucleatum, certain strains of Escherichia coli, and Bacteroides fragilis, have been reported to be important for CRC development [218]. Interestingly, Kang et al. demonstrated that APC ^Min/+^ mice receiving feces from obese human individuals were much more prone to colon tumor formation, accompanied by enrichment in pro-inflammatory pathways, including TNF signaling, cytokine–cytokine receptor interaction, toll-like signaling, and chemokine signaling, as well as induction of oncogenic signaling pathways, such as PI3K/AKT and AMPK, compared to control mice receiving feces from normal human donors [219]. The authors observed an increased abundance of Alistipes finegoldii and a decreased abundance of Bacteroides vulgatus and Akkermansia muciniphila compared with the controls [219]. Additionally, they revealed the cancer-promoting effect of Alistipes finegoldii in CRC cells and the tumor-suppressive effect of Bacteroides vulgatus and Akkermansia muciniphila [219]. From a therapeutic perspective, supplementation with *B. vulgatus* and *A. muciniphila* may be a novel strategic tool for preventing and treating obesity-related CRC [219]. Intestinal microbiome imbalance induced by a high-fat diet enhances carcinogenic pathways, impairs the tumor immune microenvironment, causes DNA damage, as well as reduces the number of dendritic cells, and SCFAs further contributing to CRC progression and worsening [217].

## 7. Targeting Obesity for Lowering Cancer Risk

CRC has been considered a sign of socioeconomic development with a Westernized lifestyle, especially high-calorie food intake and reduced physical activity, which are linked to overweight and obesity [220]. In the last three decades (1990–2022), the global BMI has approximately increased by 173% (from 25.2 to 43.5) in overweight adults and 173% (from 7.5 to 18.9) in overweight children and adolescents (Last updated 2024.02.29) [221]. Moreover, an increasing incidence of CRC has been observed in subjects aged 15–49 years [222,223]. Meta-analyses have shown that BMI is positively associated with CRC risk [6,7]. Targeting obesity or adipose factors offers a therapeutic opportunity for obesity-related metastatic cancer. Lifestyle interventions, such as diet and exercise, as well as therapeutic interventions like bariatric surgery, and pharmacological treatment, have been proven to be useful for preventing obesity-related cancer risk.

### 7.1. Control Obesity: Dietary, Exercise and Leptin/Adiponectin Administration

The dietary-switch study from the O’Keefe group indicated that the dietary switch from high-fat to high-fiber intake significantly decreased proliferation in epithelial crypt cells of colonic mucosa by influencing the microbiota and metabolome [224]. Increased total dietary fiber intake, especially from cereals and fruits, is correlated with a significantly reduced risk of colorectal adenomas [225]. Dietary fiber has also been reported to protect patients with CRC in Asia [226].

The meta-analysis containing 126 studies shows physical activity (around 10 metabolic equivalents of energy hours per week) recommended by WHO leads to a 7% reduction in cancer risk, especially in breast cancer and CRC [227]. Exercise is accompanied by elevated levels of catecholamines (epinephrine and norepinephrine), FGF-21, and IL-6, which play important roles in controlling metabolic processes and the immune system. Exercise leads to a 60% reduction in tumor incidence and growth in tumor-bearing mice and increased epinephrine and IL-6 by exercise are responsible for NK cell mobilization and redistribution [228]. Acute IL-6 administration increases lipolysis and fatty oxidation and depletes insulin-mediated glucose uptake [229]. Infusion of recombinant human IL-6 increases anti-inflammatory effects through upregulation of IL-1 receptor agonists and IL-10. Furthermore, IL-6 upregulates plasma cortisol levels, and circulating neutrophil counts, and decreases late lymphopenia [230]. Moderate-to-vigorous exercise stimulates the sympathetic nervous system to release catecholamines which enhances lipolytic activity via activation of the β-adrenergic signaling pathway [231]. Moreover, FGF-21, an exercise-responsive factor, was significantly increased after chronic exercise compared to no exercise [232]. However, FGF-21 levels were reduced after a long-term high-fat diet. Exercise can reverse dietary obesity-related FGF-21 resistance by the upregulation of FGF receptor-1 (FGFR1) and β-Klotho (KLB)-dependent browning. Moreover, chronic exercise reduces the expression of pro-inflammatory genes including TNF-α, MCP-1, IL-1β, and F4/80 [233].

Dysregulated adipokines have been identified as diagnostic, prognostic, and therapeutic targets for obesity and its metabolic comorbidities. Daily subcutaneous injections of recombinant human leptin have been used to treat congenital leptin deficiency-linked obesity [234]. In addition, leptin is a key molecule for improving T cell responsiveness by increasing the ratio of CD4+ naive T cells to IFN-γ secretion [234]. Adiponectin also plays a crucial role in obesity-related insulin resistance. Adiponectin-deficient mice have an increased number of colon polyps under a high-fat diet, suggesting that adiponectin exerts a suppressive role in colorectal carcinogenesis [235]. Adiponectin administration and adiponectin gene therapy can improve obesity-related insulin resistance [236,237]. Administration of adiponectin-mimetic peptide ALY688 shows anti-inflammatory effects by upregulation of blood T cell counts in LPS-induced mouse model [238]. PPARγ is a transcription factor that induces the expression of adipogenic genes, including adiponectin, which is a significant mediator of PPARγ activation-regulated metabolism [239]. PPARγ agonists such as Pioglitazone and Rosiglitazone have antidiabetic effects via the upregulation of adiponectin in obese subjects [240,241]. Clinical trials of PPARγ agonists as anticancer drugs are limited. Efatutazone, also known as CS-7017, RS5444, or Inolitazone, is a novel third-generation thiazolidinedione. Efatutazone alone can inhibit anaplastic thyroid carcinoma tumor growth in nude mice via upregulation of the cell cycle kinase inhibitor p21, and Efatutazone combined with paclitaxel induces apoptosis [242]. Oral administration of Efatutazone exhibited anticancer activity in a US phase I study of advanced solid malignancies with no curative therapeutic options [243]. Efatutazone in combination with 5-Fluorouracil showed acceptable safety and stable disease in Japanese patients with CRCM [244].

### 7.2. Targeting of Microbiome Imbalance

As the gut microbiota plays a significant role in controlling body weight and inflammation, therapeutic strategies involving the gut microbiota and its derivatives, particularly SCFAs, could be employed to combat obesity [50]. The utilization of probiotics, prebiotics, and fecal microbiota transplantation to modify the composition of the gut microbiota has been widely researched [245,246,247]. Additionally, SCFAs such as propionate, butyrate, and acetate may be potential therapeutic agents to alleviate inflammation resulting from imbalances in the obesity-associated microbiome. Through direct interaction with adipose tissue via the GPR43/GPR41 receptor, systemic SCFAs can lead to a reduction in lipolysis and inflammation, an increase in leptin synthesis, browning through the stimulation of UCP1, and an increase in adipogenesis [248]. The regulatory role of microbiota metabolites in adipose tissue has been further described by Li et al., where the administration of fasting microbiota every other day in mice lacking microbiome-stimulated beiging of inguinal white adipose tissue through the microbiota metabolite acetate suggested that fasting every other day has therapeutic potential [249]. However, additional research is necessary to fully comprehend the therapeutic potential of these methods, as certain studies have reported the adverse effects associated with these treatments [50,250].

### 7.3. Targeting Adipose Tissue Thermogenesis

Increased energy expenditure during adipose tissue thermogenesis is a potential therapeutic target for treating obesity-related cancer metastasis. Several natural bioactive compounds, such as polyphenols (RSV, curcumin, rutin, luteolin, and sudachitin), terpenoids (Phytol, Menthol, Celastrol), and alkaloids (Capsaicin and Berberine), can induce adipose browning via upregulation of UCP1 and UCP1 related transcriptional factors [251]. Aerobic exercise training not only increases lipolysis but also enhances the thermogenesis of WAT and BAT, however, IL-6 is essential for exercise training and cold-induced UCP-1 expression in inguinal WAT [252]. β-ARs are expressed predominantly on adipose tissue and are involved in adipose browning. Treatment of mice with a β_3_-ARs agonist (CL 316243) increased energy expenditure and insulin levels and decreased food intake, where all effects of CL316243 were abolished in β-AR knockout mice [253]. Second-generation β_3_-ARs agonist, Mirabegron (YM178), is an FDA-approved compound for the treatment of overactive bladder symptoms. Oral administration of Mirabegron (2 mg/kg/day) significantly decreased body weight, and adiposity, and upregulated UCP-1 gene expression in mice fed a high-fat diet compared to the control group [254]. Using a murine MC-38 colon adenocarcinoma cell line and a mouse CRC subcutaneous model, it has been recently found that mirabegron strongly inhibits tumor cell proliferation in vitro and tumor formation in vivo [255]. Dapagliflozin is an inhibitor of sodium glucose cotransporter (SGLT-2), which is used to control glucose levels in type II diabetes mellitus. Recently, a clinical study showed that Dapagliflozin could decrease visceral fat and body weight in patients with diabetes [256]. Dapagliflozin administration inhibits lipogenesis and induces WAT browning by downregulating lipogenesis-related genes and upregulating thermogenesis-related genes such as UCP-1, PGC1a, CIDEA, and DIO2 in mice [257].

### 7.4. Anti-Inflammatory Adipose Stem Cells

ASCs, a rich source of adult stem cells, possess high multi-lineage potential and self-renewal capacity and have been applied in tissue repair and organ regeneration [258]. ASC transplantation promotes the proliferation of organ resident cells, downregulates pro-inflammatory cytokines, and increases the number of M2 macrophages and M2-related anti-inflammatory cytokines in COVID-19 patients with pneumonia, liver injury, and diabetic retinopathy [259,260,261]. The role of ASCs in cancer therapy is still controversial. Studies on ASCs and cancer cell fusion have shown that ASCs can trigger malignant features in different cancer types, such as cervical cancer, breast cancer, and CRC [262,263,264]. For instance, IL-6 and HGF released from adipose stem cells in vWAT promote CRC expansion; in turn, tumor cells produce more neutrophils, NGF, and NT-3, and further recruit more adipose stem cells [265]. TRAIL-expressing ASCs can decrease colitis-associated colon cancer by promoting the apoptosis of CD133+ cancer stem cells [266]. More recently, Kaçaroğlu and his colleagues have reported that TLR1-primed ACSs exert an anti-tumorigenic effect on pancreatic ductal adenocarcinoma cells [267]. These modified ACSs with anti-inflammatory features offer a new approach to cancer therapy.

## 8. Targeting Lipolysis-Related Enzymes for Cancer Therapy

### 8.1. Lipolytic Enzymes (ATGL, HSL, and MAGL)

The involvement of lipolysis-related enzymes in cancer cell progression and metastasis implies that they may act as potential therapeutic targets for cancer metastasis (Table 1).

Since ATGL is the rate-limiting enzyme in TAG hydrolysis, inhibition of ATGL activity results in reduced TAG mobilization. Atglistatin, an inhibitor of ATGL, effectively inhibits adipose tissue lipolysis, body weight, insulin resistance, and non-alcohol fatty liver in mice [268]. Inhibition of ATGL by Atglistatin reverses the ATGL-induced tumorigenesis via downregulation of the genes involved in stem cell function, mitochondrial function, and lipid metabolism in colon cancer stem cells [82]. NG-497, a selective, reversible, and non-toxic human ATGL inhibitor, targets the hydrophobic cavity containing the active sites (F69, F97, and G146) of ATGL. NG-497 reduces lipolysis-dependent respiration in liver cancer cell lines [269]. NG-497 combined with glycolysis inhibitor (AZ-PFKFB3-26) synergistically inhibits colony formation in advanced prostate cancer [270].

Inhibition of HSL by an HSL inhibitor (Hi 76-0079) results in increased DAG accumulation, but not in HSL-knockout mice [271]. Hi 76-0079 efficiently reduces lipolytic capacity in WAT explants from cachexia-induced murine colon adenocarcinoma cells [272]. Tetrahydrolipstatin (Orlistat), the inhibitor of HSL and ATGL, significantly reduced the levels of glycerol in human adipocytes treated with pancreatic cell-derived exosomes [273]. The carbamate derivative CAY10499 significantly reduces invasion in KRAS^G12D^-expressing cells by inhibition of HSL [86]. 

MAGL hydrolyzes MAG into FA and glycerol. MAGL-deficient mice show a reduction in MAG hydrolase activity, resulting in MAG accumulation in different tissues, including adipose tissue; MAGL-knockout mice a fed high-fat diet show improved glucose tolerance and insulin sensitivity [274]. Systemic administration of MAGL inhibitor (MJN110) is associated with reduced feeding in rats, which may be useful for the treatment of obesity and its comorbidities [275]. LEI-515, an orally bioavailable MAGL inhibitor, attenuates liver inflammation, necrosis, and oxidative stress in a CCI4-induced acute liver injury model and effectively suppresses chemotherapy-induced neuropathic pain [276]. JZL184) suppresses cell growth and invasion in CRC and HCC cells by inhibition of MAGL [87,89]. 

**Table 1:** Antibodies and compounds targeting lipolysis-related enzymes and FA transporters.

Type	Target	Name	Mechanism	Citation
Lipolysis-related enzymes	ATGL	Atglistatin	Atglistatin reverses the ATGL-induced tumorigenesis in colon cancer stem cells	[268]
		NG-497	Reduces lipolysis-dependent respiration in liver cancer cell lines	[269]
	HSL	Hi 76-0079	Reduces lipolytic capacity in WAT explants from cachexia-induced murine colon adenocarcinoma cells	[272]
		Orlistat	Reduced the levels of glycerol in human adipocytes treated with pancreas cells-derived exosome	[273]
		CAY10499	Reduces invasion in KRAS^G12D^-expressing cells by inhibition of HSL	[86]
	MAGL	MJN110	Reduces feeding in rat	[275]
		LEI-515	shows an effective suppression in chemotherapy-induced neuropathic pain	[276]
		JEL-184	Suppresses cell growth and invasion in CRC and HCC cells	[87,89]
FA transporters	CD36	SAB	Decreases vWAT deposit and improves insulin resistance in CD36-expressing mice	[277]
		Monoclonal antibody JC63.1	Inhibits pro-metastatic effect of CAFs in CRCM by inhibition CD36-mediated metastases	[191]
		Monoclonal antibody IG04	Promotes sphere formation, stem cell frequency and apoptosis in CD36-expressing glioblastoma cells	[278]
		Nobiletin and its derivatives	Shows an anti-cancer effect through regulation of cell cycle, apoptosis, and inflammation in CRC cell lines	[279,280,281]
		Antagonist (SSO)	Inhibits cell migration in hepatocellular carcinoma and reduces cell proliferation in CRC	[282]
	FABP4	BMS309403	Alleviates severe atherosclerosis and type II diabetes	[283]
		Monoclonal antibody CA33	increases insulin sensitivity and gluco metabolism; reduces fat mass and liver steatosis	[284]
		Monoclonal antibody 2E4	Improves glucose tolerance, metabolic responses and reduces pro-inflammatory effects	[285]
		Monoclonal antibody V9	Inhibits tumor growth and metastasis in breast cancer	[286]
	FATP1	Lipofermate	Sensitizes breast and ovarian cancer cells to oncolytic virus	[287]
	FATP2	Grassofermata (CB5)	Inhibits palmitate-mediated lipid accumulation and apoptosis in CRC and liver cancer cell lines	[288]

### 8.2. Lipid Trafficking Proteins (CD36, FABPs and FATPs)

Upregulated lipid trafficking proteins are observed in adipose tissue as well as in different cancer types, including CRC. Increased lipid uptake fuels tumor growth via increased lipogenesis, oxidation, and lipolysis. Inhibition of lipid transporter proteins not only reduces vWAT deposits and improves insulin resistance in obese mice, but also reduces ovarian metastasis and CRC metastasis [91,277,289]. Targeting lipid trafficking proteins is a promising therapeutic strategy for obesity and cancer treatment (Table 1).

Salvionolic acid B (SAB) decreases vWAT and improves insulin resistance in WT obese mice, but not in CD36-depleted mice, suggesting that SAB can specifically inhibit CD36 [277]. CD36 monoclonal antibody (JC63.1) inhibits the pro-metastatic effect of CAFs in CRCM by inhibiting CD36-mediated metastases [191]. Intravascular injection with CD36 monoclonal antibody (IG04) significantly inhibits colon cancer tumor volume in human CD36-expressing mice compared to the control group [278]. CD36 antagonist, Nobiletin (NOB) and its derivatives, including 5-Demethylnobiletin (5-DMN) and NOB-metabolites, show an anti-cancer effect by regulation of cell cycle, apoptosis, and inflammation in CRC cell lines [279,280,281]. Sulfo-N-succinimidyl oleate (SSO), another CD36 antagonist, reduces proliferation in CRC cells and tumor growth in xenografts, upregulates the epithelial marker E-cadherin, and inhibits cell migration in HCC [282].

FABP4, an intracellular protein, is highly expressed in adipose tissue cell types, such as adipocytes and macrophages. High plasma levels of FABP4 occur upon the lipase activity and are regulated by elevated circulating lipids, supporting that FABP4, as an adipokine, acts as a hormone [290]. Oral administration of a FABP4 inhibitor (BMS309403) effectively alleviated severe atherosclerosis and type II diabetes in mice [283]. Moreover, the monoclonal antibodies CA33 and 2E4 against FABP4 can bind to recombinant/native FABP4. CA33 increases insulin sensitivity and glucose metabolism and reduces fat mass and liver steatosis in obese mice, whereas the effects of CA33 were abolished in FABP4-deficient mice [284]. Oral administration of 2E4 improves glucose tolerance and metabolic responses and reduces pro-inflammatory effects in obese mice [285]. Humanized V9 monoclonal antibody targeting FABP4 can inhibit tumor growth and metastasis in breast cancer [286]. Cobimetinib, the MEK inhibitor, suppresses cell proliferation and induces apoptosis in colorectal cancer cell lines and was recently discovered as a novel FABP4 inhibitor using molecular docking-based screening [291,292].

FATP1 and FATP4 are highly expressed in insulin-sensitive tissues, such as adipose tissue and the intestine. Knockdown of both FATP 1 and 4 in adipocytes results in reduced TAG accumulation [98]. FATP1 knockout mice show altered regulation of postprandial serum long-chain fatty acids (LCFAs), which alleviates diet-induced obesity and insulin desensitization [95]. FATP4-null mice show a neonatal lethal due to a perinatal dermopathy [293]. Modified FATP4-null mice with improved skin function display significantly increased TAG and FA contents in enterocytes [294]. Pharmacological inhibition of FATP1 with FATP inhibitor (Lipofermata) depletes the transport function and reduces melanoma growth and invasion [295]. Similarly, Lipofermata is able to sensitize breast and ovarian cancer cells to oncolytic virus therapy through lipid modifying of the TME [287]. Grassofermata (CB5), a FATP2 inhibitor, effectively inhibits palmitate-mediated lipid accumulation and apoptosis in CRC and liver cancer cell lines [288].

## 9. Conclusions

Obesity is an independent risk factor for cancers, including CRC. The crosstalk between CRC cells and peritumoral WAT shapes adipose tissue into a distinct appearance, the WAT browning phenotype, which is considered a potential prognostic factor and therapeutic target. Targeting the factors and pathways involved in WAT browning may bring hope to patients with CRC and cachexia. However, the treatment of adipose tissue indiscriminately could bring counter effects in obese cancer due to the opposite concepts of treatment in obesity and cancer. Immune cell infiltration, especially macrophages, neutrophils, and T cells, are considered as prognostic markers. The ratio of the two subgroups of those immune cells has a cancer-specific or context-dependent pattern. Interestingly, highly infiltrated neutrophils have both favorable and unfavorable prognoses in a CRC cohort study [163,168,296]. Those controversial data suggest that the evaluation of neutrophils in blood or tumor tissue, different tumor stages, and different neutrophil markers, could influence the results of NLR and make the reports incomparable. Targeting immune cells by inhibiting the JAK pathway has been applied in IBD. However, the side effects of increased fat accumulation and lipid profile need to be monitored for long-term treatment.

Under obese conditions, enhanced lipolysis to fuel cancer cells is a predominant feature in adipocytes. A line of evidence shows that increased lipolysis and lipolysis process-related enzymes are required for more aggressive CRC through the accumulation of secondary lipid metabolites and FFAs for FAO to produce more ATPs. Targeting lipolysis-related enzymes reduces tumor growth; however, these studies were conducted using cell and mouse models. Based on previous studies, the therapeutic targeting of lipolysis-related enzymes has potential in CRC therapy, possibly as a combination treatment with standard therapy. However, additional extensive research, particularly clinical trials, is necessary to fully reveal the safety and efficacy of this treatment.

## Data Availability

Not applicable.

## Abbreviations

NLRP3-inflammasome	NACHT, LRR und PYD domains-containing Protein
*MAPK/ERK*	Mitogen-activated protein kinases/extracellular signal-regulated kinase
*c-JNK*	c-Jun N-terminal kinase
*JAK/STAT3*	Janus kinases/signal transducer and activator of transcription proteins
*SAPK/JNK*	Stress-activated protein kinases /Jun amino-terminal kinases
SIRPα signalling	signal regulatory protein α
T-bet	T-box expressed in T cells
GATA-3	GATA Binding Protein 3
FAK	Focal adhesion kinase
CIDEA	Cell death-inducing DNA fragmentation factor, alpha subunit-like effector A
DIO2	Iodothyronine deiodinase 2
T3^b^-hIL-15 Tg mouce	A transgenic (Tg) mouse preferentially overexpresses human IL-15 in intestinal epithelial cells by the use of T3(b)-promoter
